# Expressions of resistome is linked to the key functions and stability of active rumen microbiome

**DOI:** 10.1186/s42523-022-00189-6

**Published:** 2022-06-04

**Authors:** Tao Ma, Rahat Zaheer, Tim A. McAllister, Wei Guo, Fuyong Li, Yan Tu, Qiyu Diao, Le Luo Guan

**Affiliations:** 1 Key Laboratory of Feed Biotechnology of the Ministry of Agriculture and Rural Affairs, Institute of Feed Research of Chinese Academy of Agricultural Sciences, Beijing, 100081 China; 2 4-16F, Agriculture/Forestry Center, Department of Agricultural, Food and Nutritional Science, University of Alberta, Edmonton, AB T6G 2P5 Canada; 3 Lethbridge Research and Development Centre, Agriculture and Agri-Food Canada, Lethbridge, AB T1J 4P4 Canada; 4 State Key Laboratory of Grassland Agro-Ecosystems, International Centre of Tibetan Plateau Ecosystem Management, School of Life Sciences, Lanzhou University, Lanzhou, 730000 China

**Keywords:** Rumen, Resistome, Beef cattle, Metagenomics, Metatranscriptomics, Stability

## Abstract

**Background:**

The resistome describes the array of antibiotic resistant genes (ARGs) present within a microbial community. Recent research has documented the resistome in the rumen of ruminants and revealed that the type and abundance of ARGs could be affected by diet and/or antibiotic treatment. However, most of these studies only assessed ARGs using metagenomics, and expression of the resistome and its biological function within the microbiome remains largely unexplored.

**Results:**

We characterized the pools of ARGs (resistome) and their activities in the rumen of 48 beef cattle belonging to three breeds (Angus, Charolais, Kinsella composite hybrid), using shotgun metagenomics and metatranscriptomics. Sixty (including 20 plasmid-associated) ARGs were expressed which accounted for about 30% of the total number of ARGs (187) identified in metagenomic datasets, with *tetW* and *mefA* exhibiting the highest level of expression. In addition, the bacterial hosts of 17 expressed ARGs were identified. The active resistome was less diverse in Kinsella composite hybrid than Angus, however, expression of ARGs did not differ among breeds. Although not associated with feed efficiency, the total abundance of expressed ARGs was positively correlated with metabolic pathways and ‘attenuation values’ (a measurement of stability) of the active rumen microbiome, suggesting that ARGs expression influences the stability and functionality of the rumen microbiome. Moreover, *Ruminococcus* spp.*, Prevotella ruminicola*, *Muribaculaceae* spp*. and Collinsella aerofaciens* were all identified as hosts of expressed ARGs, possibly promoting the dominance of these carbohydrate degraders within the rumen microbiome.

**Conclusions:**

Findings from this study provide new insight into the active rumen resistome in vivo, which may inform strategies to limit the spread of ubiquitously found ARGs from the rumen to the broader environment without negatively impacting the key functional outcomes of the rumen microbiome.

**Supplementary Information:**

The online version contains supplementary material available at 10.1186/s42523-022-00189-6.

## Background

Antimicrobials have been widely used in food producing animals since the 1950s to enhance feed efficiency, accelerate growth, and control infectious diseases [1]. It is estimated that antimicrobials used to prevent/treat disease and/or promote growth in chickens, pigs, and cattle will increase from 63,151 tonnes in 2010 to 105,596 tonnes in 2030 [2]. Antimicrobial consumption or administration in livestock has been proposed to select for antimicrobial resistant bacteria within the digestive tract of livestock and in aquatic/soil environments [3, 4]. The development of antimicrobial resistant bacteria in food-producing animals not only reduces the therapeutic efficacy of antimicrobials against infectious disease, but also selects for reservoirs of antimicrobial resistant genes (ARGs) that could be transferred to bacteria and infect humans via the food production chain or through the environment [5–7]. Therefore, reducing antimicrobial resistance and preventing antimicrobial residues from entering the food production chain is a priority for the livestock sectors to address food security, food safety, and public health concerns [8].

In fact, antimicrobial resistance (AMR) in bacteria is an ancient phenomenon and was present long before the widespread clinical and agricultural use of antimicrobials [9]. Numerous ARGs encode for resistance to an array of antimicrobials that microbes produce to compete and survive within complex ecological systems [10]. Recent research has documented the resistome in the rumen of ruminants including beef cattle [11–13], dairy cattle [13, 14], and sheep [15], and revealed that the profiles and abundance of ARGs within the resistome can be affected by diet [12] and/or antibiotic treatment [11]. However, most of these studies only assessed these profiles at a genomic level using metagenomics and rumen samples collected from a limited number of animals. Compared with the mere presence of a gene, its expression is a better proxy for gauging functional activity within biological ecosystems [16]. A recent study assessed the resistome within wastewater at both metagenomic and metatranscripomic levels and revealed that the abundance of ARGs and their transcripts were not highly correlated [17]. However, the extent to which ARGs are expressed in the gut of mammals such as rumen of cattle is largely unknown, especially when it is not under the selective pressure of antimicrobials.

In this study we assessed the presence (metagenomic profiling) and expression (metatranscriptomic profiling) of ARGs in the rumen of 48 beef steers raised without antimicrobials used in human medicine. To date, read-based and assembly-based approaches are two main methods for profiling the resistome, with no consensus on a single approach, as both have trade-offs [18]. Therefore, we applied both approaches in this study with the aim to comprehensively describe the ARGs profiles within resistomes as well as their expression in the rumen. We hypothesized that (1) rumen resistome may be affected by host factors such as breed; (2) not all ARGs are expressed, and those expressed may play a role in the function or stability of the rumen microbiome; (3) expression of ARGs could be associated with feed efficiency in beef cattle, as we previously reported that active rumen microbiome was linked to feed efficiency in beef cattle [19].

## Results

### Analysis of the rumen metagenomic datasets

The average number of metagenomic reads was higher in KC (59,835,594) than CH (49,746,190) (*P* = 0.009, *d* = 0.40; Additional file 1: Table S1). The proportion of metagenomic reads aligned to bovine genome was 0.13% ± 0.004% (average ± standard deviation), which did not differ among breeds (*P* = 0.134, *d* = 0.52). The average number of ARG-like reads (KC: 54,385; AN: 47,319; CH: 49,880) and their proportion of total metagenomic reads (KC: 0.090; AN: 0.088; CH: 0.098) did not differ among breeds (*P* = 0.407, *d* = 0.15 and *P* = 0.749, *d* = 0.40; Additional file 1: Table S1). After metagenomic assembly, a total of 2,776,208 contigs with an average length of 3515 bp (max 446,148 bp) and a N50 length of 6065 bp were generated. An average of 70.4%, 78.4%, and 75.2% of reads were mapped to their assemblies for KC, AN, and CH, respectively. The proportion of mapped metagenomic reads was higher in AN than KC (*P* = 0.002, *d* = 0.26). The average number of metagenomic contigs (*P* < 0.001; *d* = 0.06) and ARG-like contigs was higher (*P* < 0.001; *d* = 0.26), while the proportion of ARG-like contigs was lower (*P* < 0.001; *d* = 0.20) in KC compared with the other two breeds (Additional file 1: Table S1).

### Profiles of ARGs in the rumen microbiome and plasmids

In total, we identified 183 individual ARGs belonging to 18 classes using a read-based approach, and 104 individual ARGs belonging to 16 classes using assembly-based methods (Table 1). The abundances (calculated as reads/contigs per million of total reads/contigs) of individual ARGs identified using the two approaches are reported in Additional file 1: Table S2 and S3. Eighty-six ARGs were identified using both approaches (Additional file 2: Fig. S1a), and most were more often identified using read- vs assembly-based approach (Additional file 2: Fig. S1b). Of the 17 unique ARGs identified using an assembly-based approach, most were only identified in less than 5 samples except for *vanU* (Additional file 2: Fig. S1c). Based on these findings, we concluded that results from a read-based approach were more comprehensive and as a result this approach was used for downstream analysis.

**Table 1:** Number of individual ARGs identified using read- and assembly-based approaches in 48 rumen samples of beef steers

ARG classes	Metagenomic profiling		Metatranscriptomic profiling	
	Read-based	Assembly-based	Read-based	Assembly-based
Total	183	103	60	37
Aminoglycoside	18	10	5	1
Bacitracin	3	2	1	2
Beta-lactam	29	7	9	1
Bleomycin	1	–	–	–
Carbomycin	1	1	–	1
Chloramphenicol	5	3	–	–
Fosfomycin	1	1	–	–
Fosmidomycin	1	1	–	–
Kasugamycin	–	1	–	–
MLS	29	16	10	3
MDR	42	20	13	9
Polymyxin	1	–	1	–
Quinolone	2	1	–	–
Rifamycin	2	1	–	–
Sulfonamide	2	3	1	1
Tetracenomycin	1	16	–	–
Tetracycline	23	–	14	13
Trimethoprim	–	2	–	–
Unclassified	7	3	2	2
Vancomycin	15	15	4	4

*MLS* macrolides-lincosamides-streptogramins; *MDR* multi-drug resistant

**Fig. 1:**
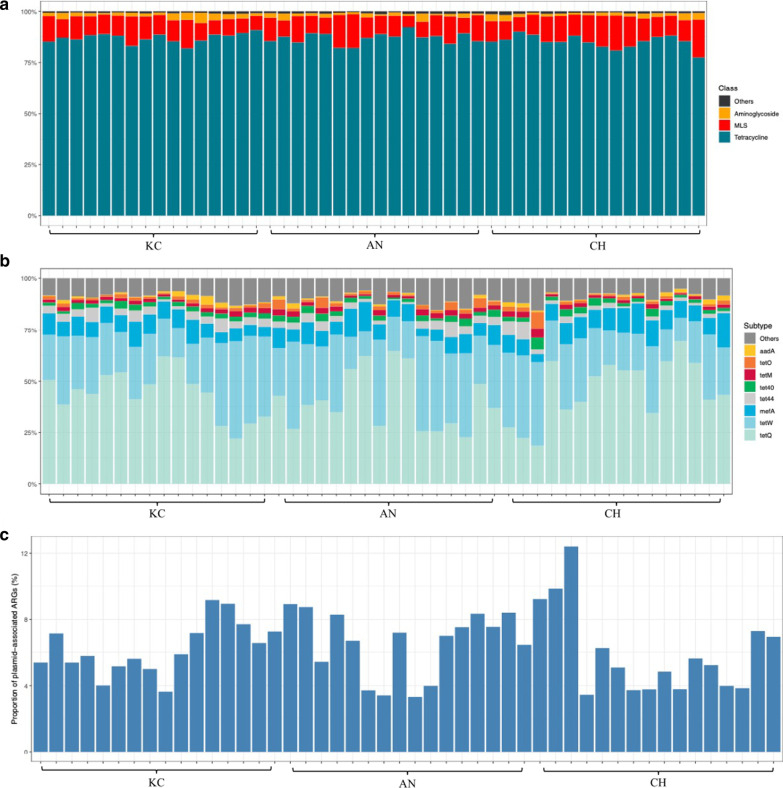
Profiles of resistome in the rumen of 48 beef cattle. **a** The rumen resistome is predominant by ARGs belonging to tetracycline, MLS, and aminoglycoside classes, and ‘others’ include other 15 classes. **b** Proportion of 8 predominant ARGs and ‘others’ include other 179 individual ARGs. **c** Proportion of plasmid-associated ARGs in the total ARGs. ARG, antimicrobial resistant gene; MLS, macrolide-lincosamide-streptogramin

Tetracycline, macrolide-lincosamide-streptogramin (MLS), and aminoglycoside classes accounted for the majority of ARGs within all samples (Fig. 1a). Eight ARGs belonging to tetracycline, MLS, and aminoglycoside classes were identified in all samples, and represented 90% of the abundance of ARGs (Fig. 1b). The PCR analysis confirmed the prevalence of *tetQ*, *tetW*, and *mefA* in extracted DNA (Additional file 2: Fig. S2). We further found that 90 ARGs, representing 15 classes were plasmid-associated (Additional file 1: Table S4), which accounted for 6.25% ± 0.30% of total ARGs (Fig. 1c). Similarly, tetracycline, aminoglycoside, and MLS were the most abundant classes (Additional file 2: Fig. S3a), and 7 ARGs (*tetW*, *aadA*, *tetO*, *tet44*, *tetM*, *vatB*, and *tet32*) accounted for 85% of the total plasmid-associated ARGs (Additional file 2: Fig. S3b).

### Analysis of the rumen metatranscriptomic datasets

The average number of metatranscriptomic reads (KC: 6,277,349; AN: 5,649,606; CH: 5,464,699), ARG-like transcripts (KC: 308; AN: 324; CH: 259) and their proportion of total metagenomic reads (KC: 0.005; AN: 0.006; CH: 0.005) was not different among breeds (*P* = 0.161, *d* = 0.55; *P* = 0.442, *d* = 0.10; *P* = 0.668, *d* = 0.44, respectively; Additional file 1: Table S5). The proportion of metatranscriptomic reads aligned to bovine genome was 0.05% ± 0.0008%, which did not differ among breeds (*P* = 0.937, *d* = 0.18). After the assembly of the metatranscriptome data, a total of 28,005 contigs with an average length of 1611 bp (max 9788 bp) and a N50 length of 1520 bp were generated. Mapped reads did not differ (*P* = 0.401, *d* = 0.01) among breeds, with an average of 82.6%, 84.0%, and 84.0% of reads for KC, AN, and CH, respectively. The average number of metatranscriptomic contigs (*P* = 0.065, *d* = 0.44) and ARG-like contigs (*P* = 0.062, *d* = 0.45) tended to be higher in AN than CH (Additional file 1: Table S5). The proportion of ARG-like metatranscriptomic contigs of total metatranscriptomic contigs did not differ (*P* = 0.591, *d* = 0.05) among breeds and was 0.145% ± 0.046% (SD), 0.159% ± 0.038%, and 0.155% ± 0.035% for KC, AN, and CH respectively (Additional file 1: Table S5).

### Profiles of expressed ARGs in the active rumen microbiome and plasmids

Sixty and 37 expressed ARGs belonging to 10 classes were identified using read- and assembly-based approaches, respectively (Table 1). Notably, the same number (60) of expressed ARGs were identified using either the SARG database or the ARG-like sequences identified in metagenomic datasets as the reference database. Abundances of expressed ARGs identified using these two approaches are shown in Additional file 1: Table S6 and S7. Twenty-three expressed ARGs were identified using both approaches (Additional file 2: Fig. S4a), with more being identified in samples using read- than assembly-based approaches (Additional file 2: Fig. S4b). Of the 14 unique expressed ARGs identified using the assembly-based method, only *tetP* were expressed in more than half of the samples (Additional file 2: Fig. S4c). Consequently, we used the read-based approach in subsequent downstream analysis of expressed ARGs.

**Fig. 2:**
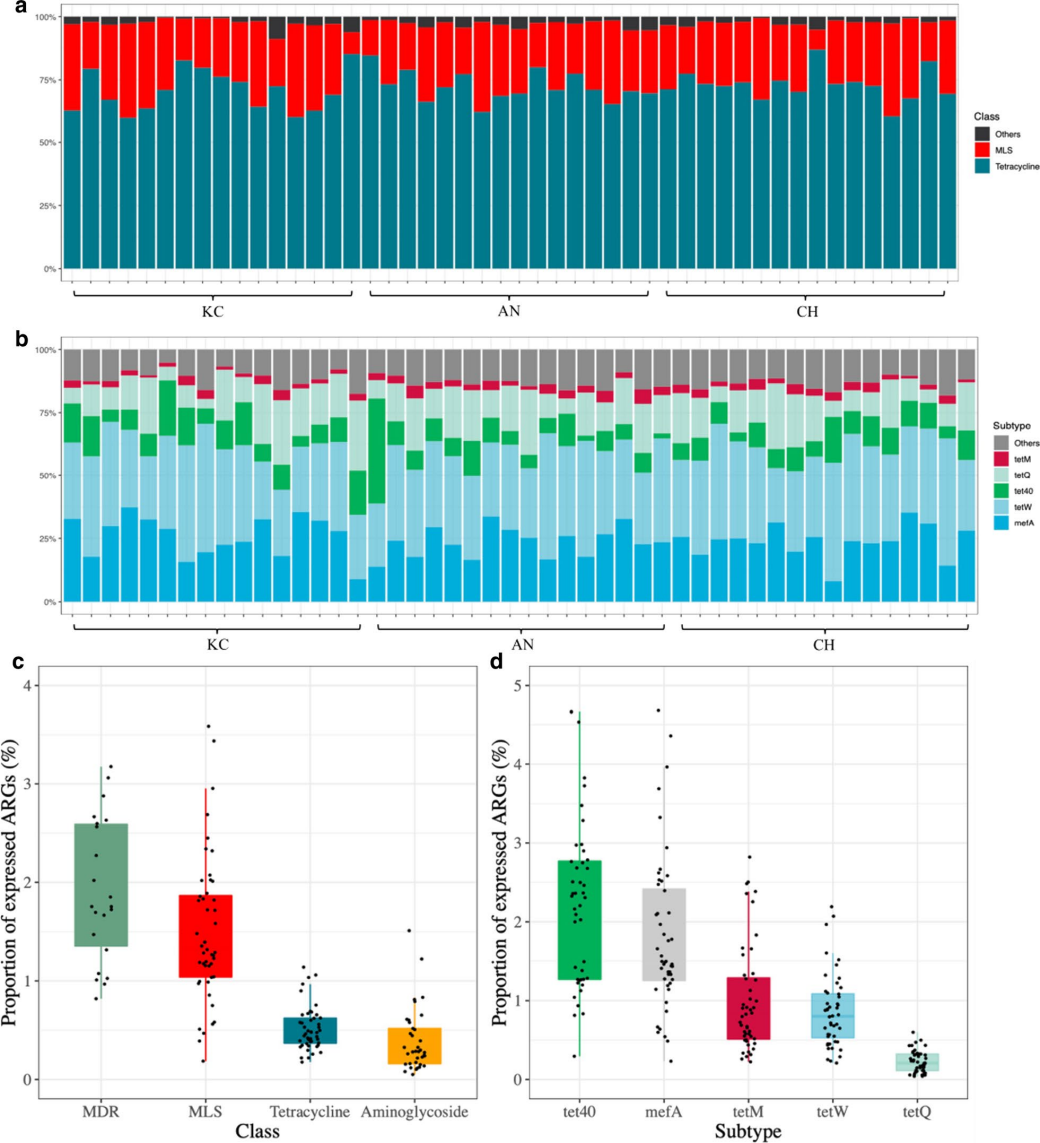
Profiles of active resistome in the rumen of 48 beef cattle. **a** The active rumen resistome is predominant by ARGs belonging to tetracycline and MLS classes, and ‘others’ include other 8 classes. **b** Proportion of 5 predominant expressed ARGs and ‘others’ include other 55 individual expressed ARGs. **c** Proportion of major expressed ARG classes. **d** Proportion of major expressed ARGs. ARG, antimicrobial resistant gene; MDR, multidrug; MLS, macrolide-lincosamide-streptogramin

Tetracycline (*tetW*, *tet40*, *tetQ*, *tetM*) and MLS (*mefA*) classes made up the majority of expressed ARGs from all samples (Fig. 2a and b). Indeed, only 0.61% ± 0.03% (calculated as the number of ARG-like reads of a sample identified in metatranscriptomic data/number of ARG-like reads of the same sample identified in metagenomic data) of ARGs were expressed, with multidrug (MDR, n = 28), MLS (n = 48), tetracycline (n = 48), and aminoglycoside (n = 37) classes being most prevalent (Fig. 2c). Of the expressed ARGs, *tet40*, *mefA*, *tetM*, *tetW*, and *tetQ* were the most prevalent in all samples (Fig. 2d). The PCR analysis confirmed the expression of *tetQ*, *tetW*, and *mefA* in the cDNA of the majority of samples (Additional file 2: Fig. S2). Twenty of the expressed ARGs were plasmids-associated and represented 7 different classes (Additional file 1: Table S8). Transcripts associated with tetracycline ARGs were the most prevalent (Additional file 2: Fig. S5a), with plasmid-associated *tetW* being identified in all samples (Additional file 2: Fig. S5b).

### Effect of breed on diversity and abundances of ARGs and expressed ARGs in the rumen microbiome

**Fig. 3:**
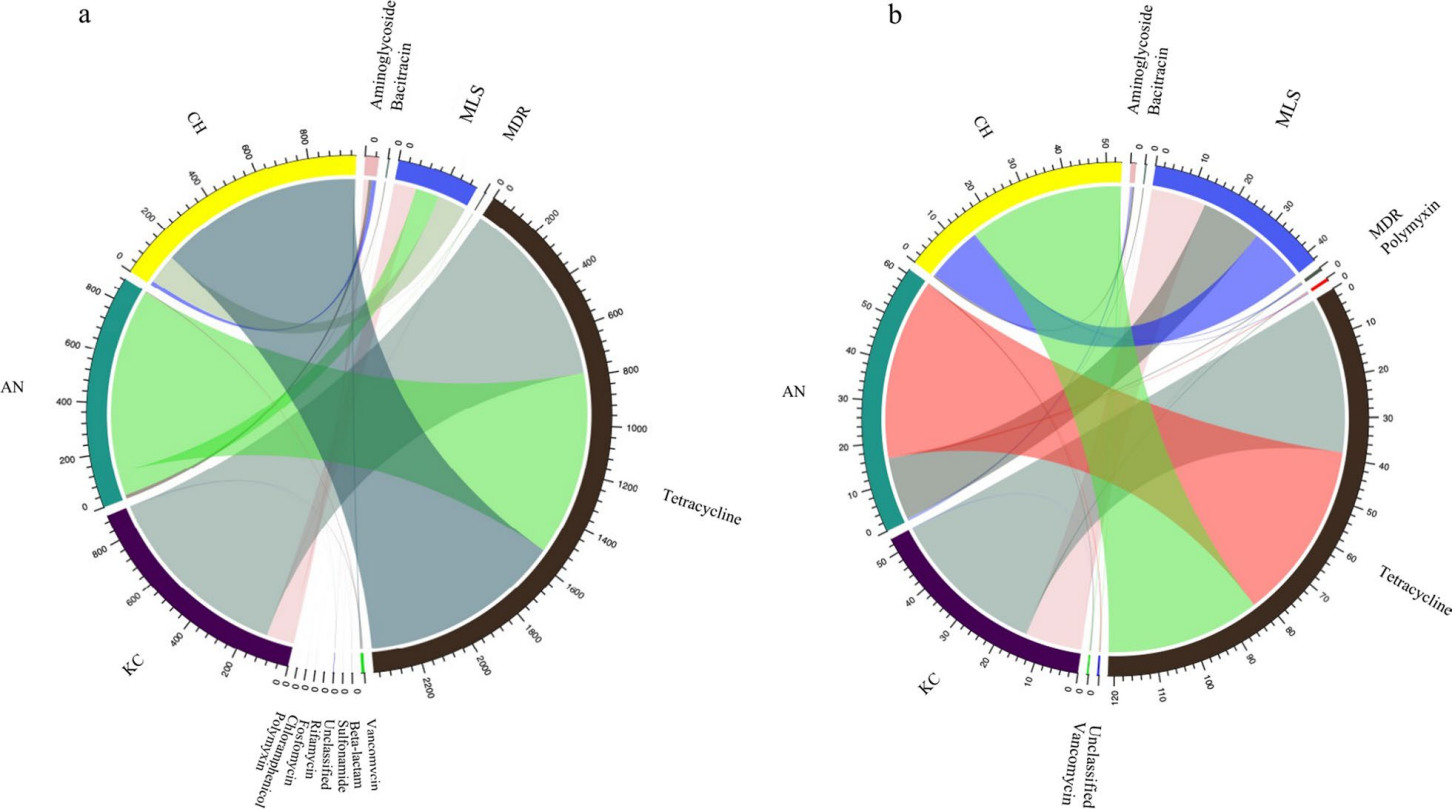
Abundance (normalized into reads per million of reads) of classes of ARGs and ARG transcripts in the rumen of KC, CH, and AN beef steers. **a** Abundance of 13 classes of ARGs. **b** Abundance of 8 classes of expressed ARGs. ARG, antimicrobial resistant gene; KC, Kinsella composite hybrid; AN, Angus; CH, Charolais; MLS, macrolide-lincosamide-streptogramin; MDR, multidrug

**Fig. 4:**
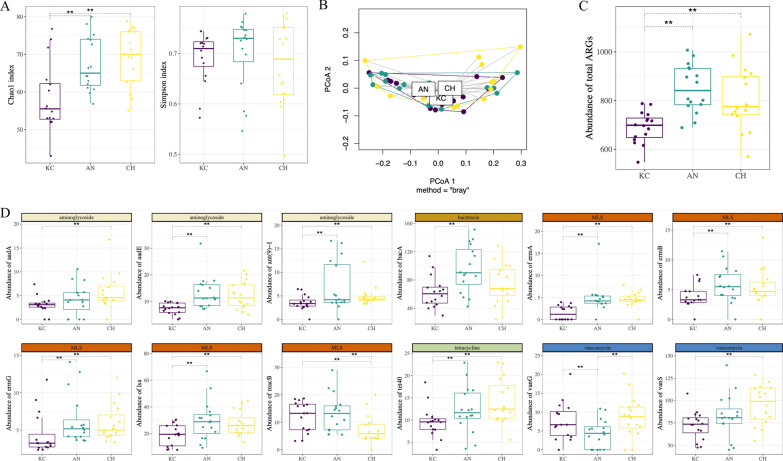
Difference in diversity annd abundance (normalized into reads per million of reads) of ARGs in the rumen of KC (in purple), CH (in green), and AN (in yellow) beef steers. **a** Difference in Chao1 index and Simpson’s index among breeds. **b** PCoA based on Bray–Curtis distance. **c** Difference in the abundance of total ARGs among breeds. **d** Difference in the abundance of 12 ARGs belonging to aminoglycoside, bacitracin, MLS, tetracycline, and vancomycin classes among breeds. ARG, antimicrobial resistant gene; KC, Kinsella composite hybrid; AN, Angus; CH, Charolais; MLS, macrolide-lincosamide-streptogramin; MDR, multidrug. PCoA, principal coordinate analysis. **P < 0.05 by Kruskal–Wallis test followed by multiple pairwise comparisons using Dunn’s test with Benjamini–Hochberg method for false discovery rate control

The ARGs belonging to tetracycline and MLS classes were predominant in the rumen, regardless of breeds (Fig. 3a). The Chao1 index of ARGs was lower in KC than AN and CH (*P* = 0.007 and 0.002, *d* = 0.24), while the Simpson’s index of ARGs did not differ among breeds (*P* = 0.512, *d* = 0.29; Fig. 4a). Principle Coordinate Analysis (PCoA) showed that the profiles of ARGs also did not separate by breed (PERMANOVA *P* = 0.241; Fig. 4b). The KC (125) harbored fewer subtypes of ARGs compared with AN (153) or CH (136) (Additional file 1: Table S9). The abundance of total ARGs in KC was also lower than that in the other two breeds (Fig. 4c). Specifically, the abundance of several ARGs belonging to aminoglycoside (*aadA*, *aadE*, *ant(9)-I*), bacitracin (*bacA*), MLS (*ermA*, *ermB*, *ermG*, *lsa*, *macB*), tetracycline (*tet40*), and vancomycin (*vanG*, *vanS*) (Fig. 4d) classes in KC were lower than in AN or CH.

The KC (64) harbored fewer subtypes of plasmid-associated ARGs compared with AN (74) or CH (67) (Additional file 1: Table S10). No difference was observed in the abundance of total plasmid-associated ARGs among breeds. The KC had a higher abundance of *aadE* as well as *vatB* than AN, and *ant(9)-I*, *aph(3″)-I*, as well as *tet44* than CH (Additional file 2: Fig. S6). In contrast, the abundance of *ermB*, *ermG*, and *lnuA* was lower in KC than the other two breeds (Additional file 2: Fig. S6).

The Chao1 index of expressed ARGs was lower in KC than AN (*P* = 0.024, *d* = 0.55), with no difference in the Simpson’s diversity among breeds (*P* = 0.541, *d* = 0.24; Additional file 2: Fig. S7a). The PCoA based on Bray–Curtis distance showed no clustering of expressed ARGs within the resistome among breeds (PERMANOVA *P* = 0.423; Additional file 2: Fig. S7b).

Metatranscriptomic analysis also showed that ARGs belonging to tetracycline and MLS classes were predominant in the expressed rumen resistome of all three breeds (Fig. 3b). A similar number of subtypes of expressed ARGs were identified in KC (44, including 26 plasmid-associated), AN (42, including 31 plasmid-associated), and CH (42, including 26 plasmid-associated), among which 30 (including 18 plasmid-associated) were identified in all breeds (Additional file 1: Table S11 and S12; Additional file 2: Fig. S7a and S7b). Breed did not impact the abundance of expressed ARGs (including plasmid-associated).

### Investigation of the bacterial hosts of expressed ARGs

**Fig. 5:**
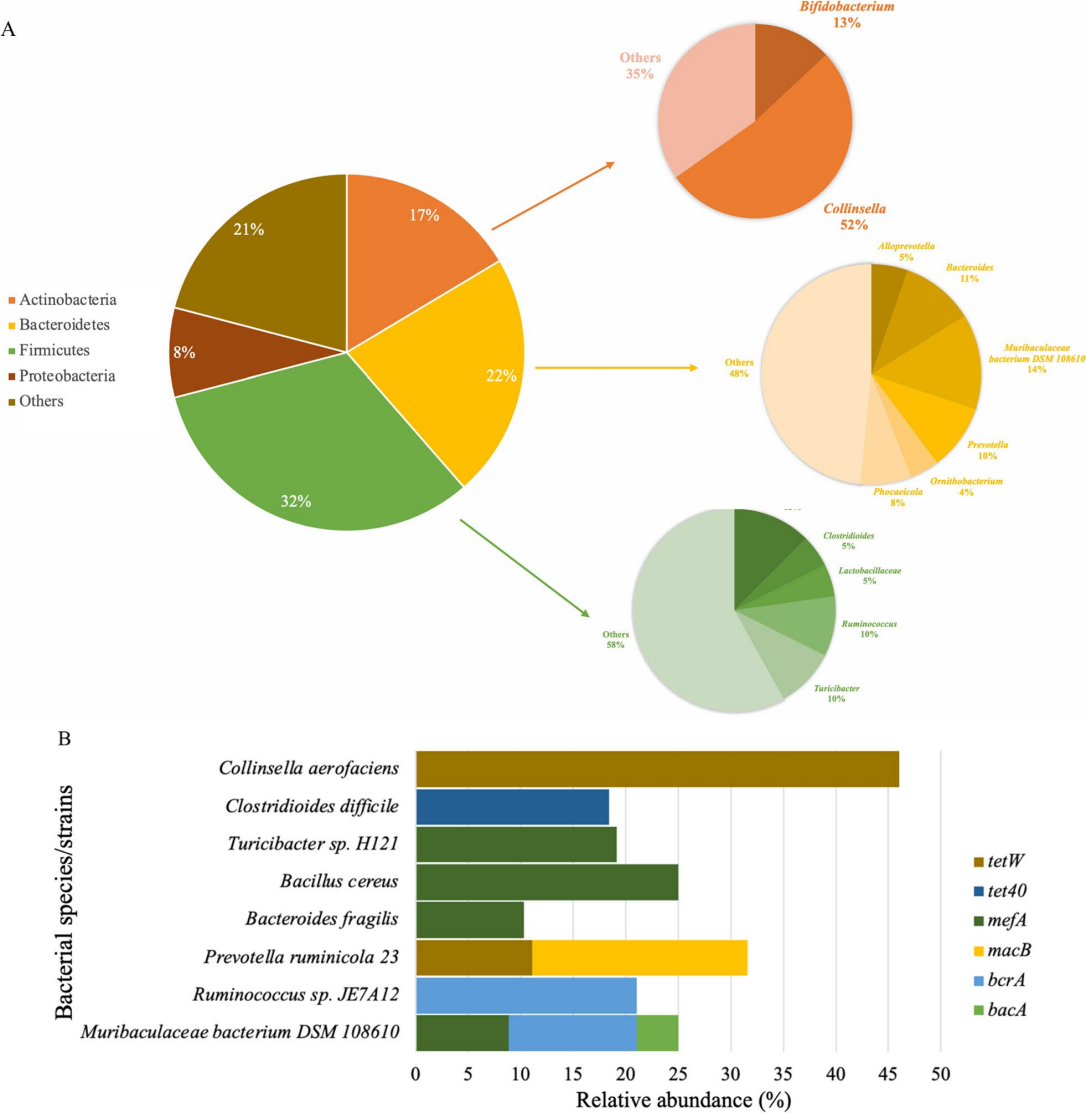
The bacterial origin of active resistome. **a** Proportion of predicted bacterial hosts of expressed ARGs at phylum level. **b** Predicted bacterial hosts of 6 expressed ARGs including *tetW*, *tet40*, *mefA*, *macB*, *bcrA*, and *bacA* and their relative abundance. ARG, antimicrobial resistant gene

The ARG-containing contigs generated by metatranscriptomic assembly were used to predict the bacterial origin of expressed ARGs. A total of 420 ARG-containing contigs were assigned to a customized reference database (see Methods) and the bacterial hosts of 342 contigs were identified as belonging to Firmicutes (32.4%), Bacteroidetes (22.1%), Actinobacteria (16.4%), and Proteobacteria (8.1%) (Fig. 5a). For the bacterial hosts identified in Actinobacteria, the genus *Collinsella* (52.2%) accounted for more than half of the abundance, followed by *Bifidobacterium* (13.0%) (Fig. 5a). *Muribaculaceae* bacterium DSM 108,610 (14.0%) was the most abundant genus in the Bacteroidetes, followed by *Bacteroides* (10.7%) and *Prevotella* (9.7%) (Fig. 5a). In the Firmicutes, *Bacillus*, *Ruminococcus*, and *Turicibacter* were the most abundant genera (Fig. 5a). The 342 assembled-genomes were found to contain 17 expressed ARGs, among which *mefA* was the most abundant, followed by *tetW* and *tetQ* (Additional file 1: Table S13). Notably, we identified 8 bacterial species/strains that could potentially express *bacA*, *bcrA*, *macB*, *mefA*, *tet40*, and *tetW* (Fig. 5b). For example, *Muribaculaceae* bacterium DSM 108,610, *Bacteroides fragilis*, *Bacillus cereus*, and *Turicibacter* sp. H121 potentially expressed *mefA*, while *Collinsella aerofaciens* and *Prevotella ruminicola* 23 potentially expressed *tetW*.

Using only the flanking regions of ARGs from the ARG-containing metatranscriptomic contigs, a total of 97 contigs were assigned to a customized reference database and the bacterial hosts were identified as belonging to Firmicutes (37.1%), Actinobacteria (25.8%), Bacteroidetes (19.6%), and Proteobacteria (4.1%) (Additional file 2: Fig. S8). For the bacterial hosts identified in Actinobacteria, the genus *Collinsella* (84.0%) accounted for more than half of the abundance, followed by *Bifidobacterium* (8.0%) (Additional file 2: Fig. S8). *Muribaculaceae* bacterium (26.3%) was the most abundant genus in the Bacteroidetes, followed by *Muribaculum* (21.0%) and *Phocaeicola* (15.8%) (Additional file 2: Fig. S8). In the Firmicutes, *Bacillus*, *Ruminococcus*, and *Turicibacter* were the most abundant genera (Additional file 2: Fig. S8). The 97 metatranscriptomic-assembled genomes were found to contain 13 expressed ARGs, among which *mefA* was the most abundant, followed by *tetW* (Additional file 1: Table S14). Notably, we identified 6 bacterial genera/species (*Muribaculaceae*, *Bacteroides fragilis*, *Ruminococcus* spp, *Bacillus cereus*, and *Turicibacter* spp., and *Collinsella aerofaciens*) that could potentially express *mefA* or *tetW* (Additional file 1: Table S14). In summary, bacterial species that were identified using ARG flanks were all included in the list of species identified using full-length contigs.

### Functional potentials of expressed ARGs in the rumen microbiome

**Fig. 6:**
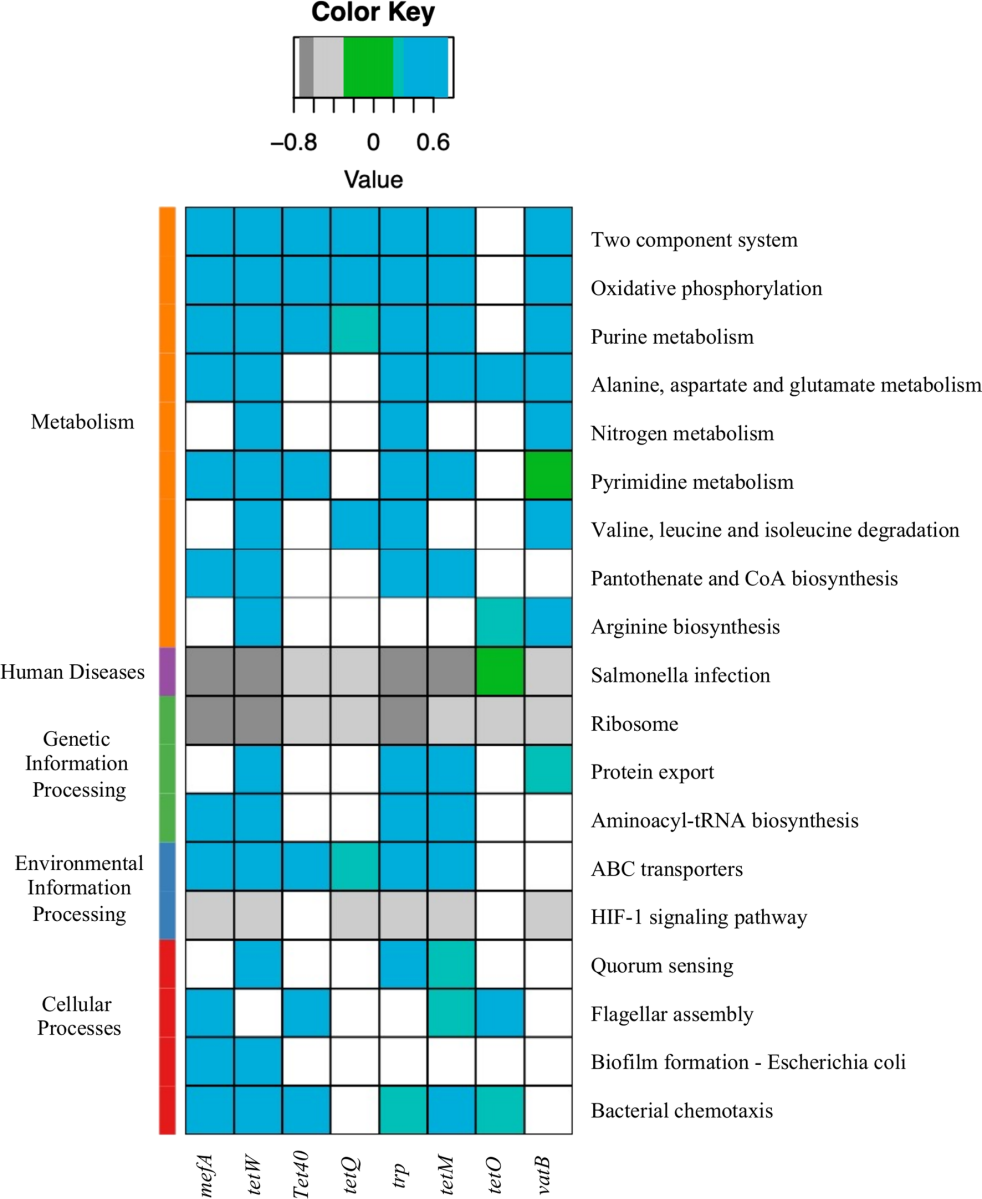
Heatmap showing the Spearman correlations between the abundance (normalized into reads per million) of KEGG pathways and the abundance of 8 subtypes of expressed ARGs. ARG, antimicrobial resistant gene; *trp*, tetracycline resistance protein

We first investigated if expressed ARGs contribute to the annotated function of the active rumen microbiome. Based on Spearman correlation analysis, we found significant correlations between the abundance of 19 KEGG pathways mainly related to ‘Metabolism’, ‘Cellular Processes’ as well as ‘Genetic Information Processing’ and the abundance of 8 subtypes of expressed ARGs, including *mefA*, *tetW*, *tet40*, *tetO*, *tetM*, tetracycline resistance protein (*trp*), *tetO*, and *vatB* (Fig. 6). Notably, positive correlations were observed between the abundance of most subtypes of expressed ARGs and ‘Metabolism’ pathways including ‘Two component system’, ‘Oxidative phosphorylation’, and ‘Purine metabolism’, while negative correlations were identified between those and ‘Salmonella infection’, ‘Ribosome’, as well as the ‘HIF-1 signaling pathway’ (Fig. 6). We then analyzed if the expression of the resistome was associated with feed efficiency in beef cattle. However, no significant correlation was observed between the abundance of total expressed ARGs or any individual ARGs and residual feed intake (RFI) or feed conversion ratio.

**Fig. 7:**
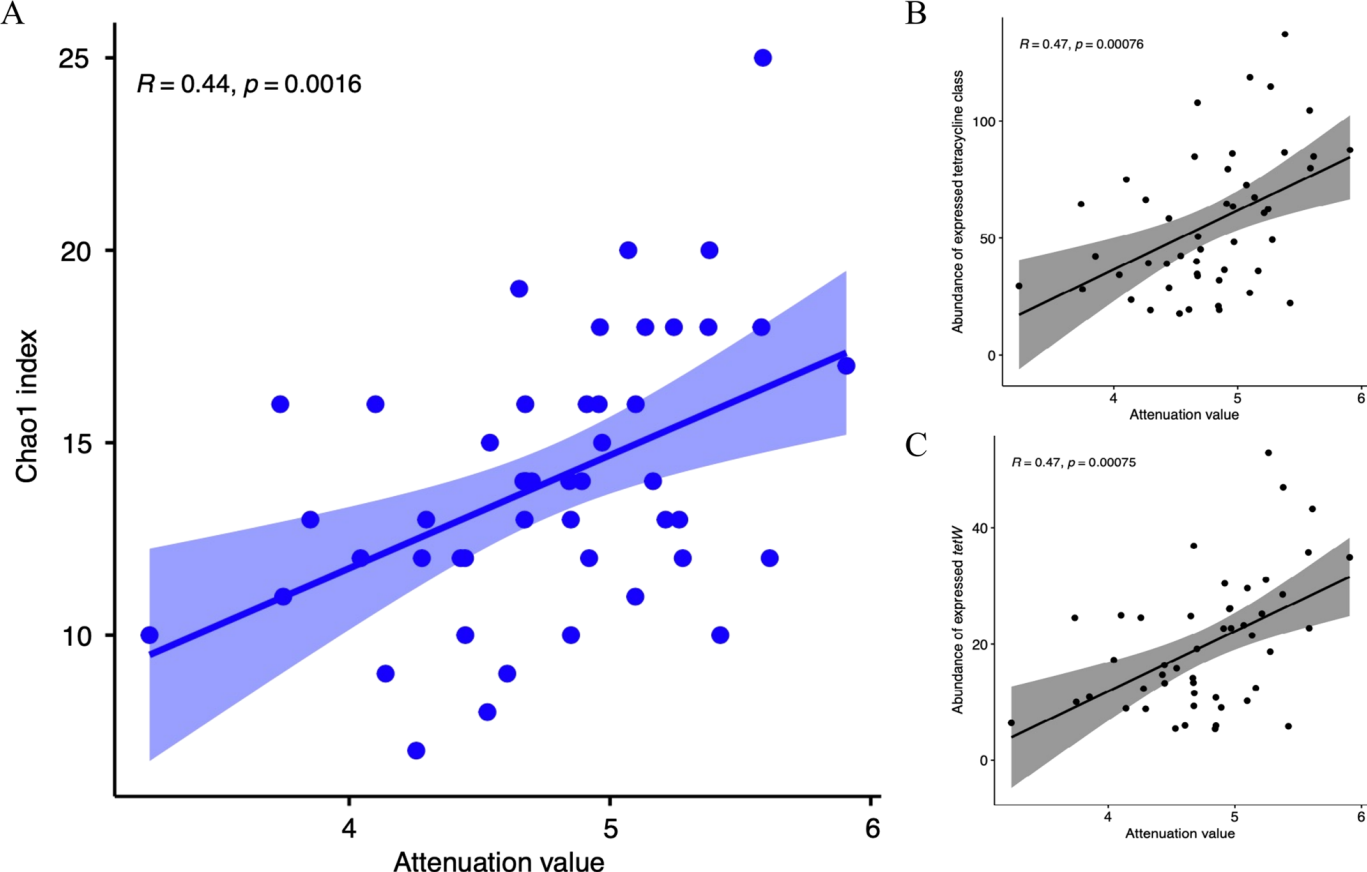
Functions of active resistome. **a** Spearman correlation between the Chao1 index of expressed ARGs and attenuation values of active rumen microbiota. **b** Spearman correlation between the abundance of expressed ARGs in tetracycline class and attenuation values of active rumen microbiota. **c** Spearman correlation between the abundance of expressed *tetW* and attenuation values of active rumen microbiota. ARG, antimicrobial resistant gene

To test our hypothesis that an active resistome may play a role in conferring stability to the rumen microbiome, we calculated the attenuation value, a measurement of microbial stability. The Chao1 index of the active resistome was positively correlated with the attenuation value (*rho* = 0.46, *P* < 0.05; Fig. 7a). In addition, we also found that the abundance of expressed tetracycline class (*rho* = 0.47, *P* < 0.05; Fig. 7b) and *tetW* (*rho* = 0.47, *P* < 0.05; Fig. 7c) was positively correlated with the attenuation values of the active rumen microbiome, indicating that it may also contribute to functional stability.

## Discussion

Metagenomic analysis revealed that the abundance of ARGs in the rumen of beef steers that did not receive any antimicrobials other than monensin, was predominated by tetracycline, MLS, and aminoglycosides. We detected broader ARG profiles using a read-based approach (187 ARGs belonging to 18 classes) in the rumen, than that observed in a previous study where ARGs in beef cattle receiving monensin and tylosin were estimate using an assembly-based approach [11]. These researchers failed to detect aminoglycoside or β-lactam ARGs in any of the 5 ruminal samples examined. While Auffret et al. [12] detected macrolide, chloramphenicol, β-lactam, and aminoglycoside ARGs in the rumen of antimicrobial-free beef cattle under similar feeding condition to our study, they did not detect genes conferring resistance to vancomycin. More recently, Xue et al. [14] described a resistome containing 26 classes of ARGs using rumen digesta samples collected from 49 dairy cows that did not receive antimicrobials, with tetracycline being the most predominant, followed by glycopeptide and fluoroquinolone ARGs. The variation of ARGs profiles detected among studies could be due to difference in animal, environment, diet, antimicrobial use (AMU) as well as metagenome data nature and bioinformatic resources/tools. In addition, the detected ARGs are similar to those reported in fecal samples of beef cattle administered in feed antimicrobials (ionophores, chlortetracycline, or tylosin) [20] or not [21]. This suggests that the profiles of ARGs in the rumen are consistent with those found in feces. It should be noted that tetracycline and macrolide are the most frequently used antimicrobials in beef industry in Canada [22]. Such a practice could have resulted in these ARGs being the key components of the core resistome in the rumen, even in the absence of direct selective pressure as result of AMU.

Considering that the presence of genes does not necessarily directly correlate with their expression in biological systems, direct measurement of transcripts based on metatranscriptomics is an important complementary approach to describing the functionality of microbiomes. In our study, the abundance of expressed ARGs was about 30% of the whole resistome, suggesting that most ARGs were not transcribed and functional in the rumen of these cattle at the time of sampling. In fact, the expression of ARGs is not directly linked to the presence of ARGs as previously shown in wastewater microbiomes [17]. The expression of *tet40*, *mefA*, *tetM, tetW*, and *tetQ* was observed in all samples, suggesting that these ARGs may constitute the ‘core’ active resistome in the rumen of the steers investigated in our study. However, it is not clear if and how this expression pattern may change if antimicrobials are administered. To our knowledge, few studies have examined the presence and expression of ARGs simultaneously in microbiomes within food-producing animals. Although Sabino et al. [23] analyzed the expression of rumen ARGs, only 15 samples were used (5 dairy cattle, 5 beef cattle, and 5 sheep) and their aim was to confirm the expression of ARGs found in 435 ruminal bacteria and archaea reference genomes in silico*.* These researchers did not link the expression of ARGs back to the presence of those ARGs in the same rumen microbome using metagenomic data. More recently, the resistome in the chicken and pig gut were analyzed using both metagenomic and metatranscriptomic data, but only 6 fecal samples were used to define the gut resistome of each species [24]. In this regard, more effort is needed to detect and validate our findings based on both metagenomic and metatranscriptomic analysis. In addition, it should be noted that the average insert size varied between the DNA and cDNA libraries due to the fact that with an average gene length of ~ 1 kb in prokaryotes, and with selective expression, the overall transcriptome is more fragmented with essentially smaller average fragment size as compared to genomic DNA fragments that are generated due to DNA shearing. This difference may potentially lead to smaller average insert size of the cDNA-based library compared to a metagenomics DNA-based library. Smaller insert sizes may also impact the quality of reads and the downstream analysis of functional profiles (such as resistome) of Illumina sequencing output [25, 26]. Therefore, comparing communities across multiple experiments may require additional covariates (e.g., library preparation technique) in statistical design to account for these known differences and avoid potential biases. It should also be noted that there is still a lack of direct comparison of metagenomic and metatranscriptomic datasets from the same sample, and our results advanced the knowledge on what are key factors (e.g., sequencing depth, N50 length, library size) in affecting the data analysis outcomes as a whole.

Plasmids are mobile genetic elements that are abundant in the bacterial populations of bovine rumen [27], and play a major role in the spread of AMR through horizontal gene transfer [28]. Plasmid-mediated transfer of ARGs is the most common route for their acquisition by bacteria [29]. Metagenomic approaches have been used to characterize plasmid encoded ARGs in several non-biological habitats such as activated sludge [30, 31] as well as in the human gut [32]. However, the profiles and expressions of plasmid-associated ARGs have not been examined in food-producing animals. The identification of *aadA* and *tetW* being the most abundant plasmid-associated ARGs and the expression of *tetW* highest among all ruminal plasmid-associated ARG transcript, suggests that plasmid associated ARGs should be a focus of investigations on the impact of AMU in livestock on the resistome. The *aadA* gene has been detected on IncA/C2 plasmids in both *Escherichia coli* and *Salmonella enteric* [33]. More specifically, *aadA14* was first identified as plasmid borne in a bovine respiratory disease-associated pathogen, *Pasteurella multocida* [34]. It has been reported that many of the tetracycline resistant genes are associated with mobile plasmids [35], among which *tetW* has been proven to be transferable among the ruminal bacteria, i.e., *Butyrivibrio fibrosolvens*, *Selemonas ruminitanium*, and *Mitsuokella multiacidus* [36]. It has been proven that rumen plasmidome, which represents all detectable plasmids in rumen, encodes a wide array of genes with roles in carbohydrate, protein and amino acids metabolism [27]. Therefore, we speculate that the plasmids that carry expressed ARGs likely also carry genes that play a role in fermentation. Recently, linking a wide range of bacterial plasmids to the microbiome in wastewater samples has been undertaken using the Hi-C method [37], which can link plasmids back to their bacterial host [38]. These approaches could also be applied to link plasmid-associated ARG to their rumen bacteria, which may help elucidate the contribution of plasmids to the transmission of ARGs within the rumen.

It has been reported that the prevalence and abundance of ARGs in the gut of cattle is affected by diet and host. For example, dietary transition from milk replacer to a starter diet led to alternation in the fecal resistome of dairy calves [7]. In addition, the diversity and abundance of total ARGs were higher in the rumen of beef cattle fed high concentrate than those fed high forage diet, with chloramphenicol and aminoglycoside resistance genes being predominant in forage- and concentrate-fed cattle, respectively [12]. On the other hand, several studies have shown that the gut microbiome is heavily influenced by the host animal [39–41], suggesting that host may also play a key role in shaping the rumen resistome. In this study, all beef steers were raised under the same dietary and environmental conditions, suggesting that the prevalence and expression of ARGs could have been influenced by host genetics. Contrary to the findings of Auffret et al. [11], who did not observe a breed effect on the abundance of microbiota or ARGs in the rumen of beef cattle, we observed a reduced prevalence of ARGs in the rumen of crossbred (KC) compared to purebred (AN or CH) cattle. Cohen’s *d* is a common way to measure the size of an effect, which is classified as small (*d* = 0.2), medium (*d* = 0.5), and large (*d* ≥ 0.8) [42]. Although there is significant difference in the average number of metagenomic reads and Chao1 index of ARG, only small to medium effect size was identified, suggesting that sequencing depth may not be a potential confounding factor in the comparison of the breed effect on diversity and abundance of ARGs. To our knowledge, there is no study reporting how host genetic factors may impact the gut resistome in mammalian species. In beef cattle, understanding the ‘mammalian host-resistome’ association may be a prerequisite to select breeds with high feed efficiency and a lower risk of transmission ARG into the environment.

The positive correlation between the abundance of multiple subtypes of expressed ARGs and ‘Metabolism’ pathways suggest that ARGs may play a role in regulating bacterial metabolism. Indeed, ARGs, such as those encoding for efflux pumps may have roles in regulating microbial physiology and metabolism of amino acids, fatty acids or nucleotides, in addition to their intrinsic role in antimicrobial resistance [43–45]. Bacteria belonging to *Muribaculaceae* spp. encode enzymes that degrade plant hemicellulose and pectin [46]. *Collinsella aerofaciens* has been reported to be abundant in the rumen of low-methane yield sheep [47], which could ferment a range of different carbohydrates such as starch [48]. *Prevotella ruminicola* [49] and *Ruminococcus* spp. [50, 51] are also prevalent fiber degraders within the rumen. Our findings raise the possibility that expression of ARGs could impact the relative contribution of these species to ruminal fiber and starch digestion. Knowledge of the bacterial hosts of expressed ARGs could play an essential role in designing strategies to limit the spread of ARGs via manipulation of the rumen microbiome. Despite these findings, it should be noted that a large proportion of hosts of expressed ARGs could not be taxonomically classified or could only be identified at the phylum/family level. Considering the fact that when expressed, the transcript of a gene does not contain extensive flanking regions of a gene coding sequence (cds) except for the 5′ untranslated region (5′UTR) until the transcription start site upstream of an individual gene or the first cds in an operon where the member genes of the operon are transcribed together, and a small 3′ termination (rho-dependent or independent) signal downstream of an individual gene cds or the last cds of an operon, it is challenging to predict bacterial origin from metatranscriptomic assemblies. However, for certain genomic loci in prokaryotes where gene/operon transcripts overlap and if those loci happen to carry an ARG, the resulting assembled long ARG-carrying contigs may lead to a better prediction of bacterial species. This could be the case of the 97 ARG-containing metatranscriptomic contigs identified as active bacterial hosts using only the flanking regions that were not mobile genetic elements or partial ARGs. Conversely, although metagenomic assembled ARG-carrying contigs potentially harboring contiguous sequences of the host bacteria could provide better prediction of bacterial species, ARGs present in those contigs may or may not be expressed making the functionality of ARGs within the rumen microbiome questionable. In this regard, more effort is needed to develop parallel metagenomics and metatranscriptomic approaches to identify functional ARGs and their host genomic context.

We did predict that the expression of tetracycline class, especially *tetW,* was associated with the stability of active rumen microbiota. Stability refers to the ability of a microbial community to return to its original state after facing perturbations [52]. Higher stability (higher attenuation value in our study) indicates that a microbial community is more resilient to external perturbations and its functional profile is therefore less likely to change. Our findings suggest that a rumen microbial community with higher diversity or expression of *tetW* may be more resistant to external perturbations such as use of antimicrobials or changes in diet compositions. However, as the correlation analysis is not able to imply causation, the exact role that expressed ARGs play in regulating these functions requires further investigation.

## Conclusions

We characterized ARGs profiles including the ‘active’ resistome within the rumen of beef steers that were not administered antimicrobials other than monensine. Our findings demonstrated that the presence and expression of ARGs in the rumen are not necessarily associated with AMU. Although a diversity of ARGs were found in the rumen, about 30% (60/183) were expressed, with multiple tetracycline ARG subtypes and *mefA* constituting the ‘core’ of the active rumen resistome. Individual PCR analysis further validated the prevalence and expression of the core ARGs identified using metagenomic and metatranscriptomic sequencing. We also revealed that *Ruminococcus* spp., *Prevotella ruminicola*, *Muribaculaceae* spp. and *Collinsella aerofaciens* were the primary hosts of expressed ARGs. In addition, both breed and feed efficiency exhibited a weak effect on the abundance of expressed ARGs, suggesting that in the absence of antimicrobial use, the rumen resistome is unlikely to be altered by breeding programs that select cattle based on improved feed efficiency. We also showed that the active resistome in the rumen may play a role in bacterial metabolism as well as maintaining the stability of active rumen microbiome. One potential limitation of the current study is that rumen resistome originated from cattle that only received monensin. It is unclear whether the overall rumen resistome or its expression would have been if antimicrobials such as tetracyclines or macrolides had been administered. In addition, variations in sequencing depth and library construction technique between metagenomic and metatranscritpomic datasets in our study may also impact the profiles of expressed resistome. Comparative analysis of transcriptional profiles of rumen samples originating from larger herds of beef or dairy cattle at different growth stages, raised with and without antimicrobials could provide further insight on the presence and expression of ARGs within the rumen microbiome. Considering that the N50 length for metatranscriptomic-assembled contigs were relatively short, appropriate method to identify active bacterial host of expressed ARGs based on metatranscriptomic sequencing needs further investigation. This study provides new insight into the active rumen resistome in the absence of antimicrobial selective pressure, generating information that could be used to develop strategies to limit the spread of ubiquitously found antimicrobial resistance from the rumen to the broader environment.

## Methods

### Animal experiment and sample collection

The datasets used in the current study originated from the study of Li et al. [53]. Briefly, ruminal digesta samples were collected from 48 beef steers at slaughter (range from 429 to 554 days of age, on average 502 ± 33 days of age) and snap-frozen in liquid nitrogen. The beef steers included three breeds: Kinsella composite hybrid (KC, n = 16), Angus (AN, n = 16), and Charolais (CH, n = 16). All steers were raised in the same feedlot conditions and fed the same diet, consisting of 80% barley grain, 15% barley silage, and 5% supplement. Cattle did not receive any antimicrobials other than monensin at 33 ppm in the diet, an ionophore that is not used in human medicine.

### Metagenome and metatranscriptome sequencing

Total DNA and RNA were isolated from rumen digesta of 48 beef steers using the methods of Yu and Morrison [54] and Li et al. [55], respectively. After quality and quantity checks, metagenomic libraries of the DNA were constructed using TruSeq DNA PCR-Free Library Preparation Kit (Illumina, San Diego, CA, USA) and subjected to Illumina (HiSeq 2000) sequencing. After measurement of RNA yield and quality, samples with RNA integrity number (RIN) > 7.0 were used to construct metatranscriptome libraries using the TruSeq RNA Library Prep Kit v2 (Illumina, San Diego, CA). All metagenomic and metatranscriptomic libraries (n = 16 for each breed) were sequenced (1 μg of input DNA or cDNA library) at the McGill University and Génome Québec Innovation Centre (Montréal, QC, Canada) using an Illumina HiSeq 2000 platform (100 bp paired-end sequencing of ~ 350 bp inserts for metagenome, and of ~ 140 bp cDNA fragments for metatranscriptome). Quality control (QC) of each dataset was performed as described by Li et al. [53]. Briefly, Trimmomatic (version 0.35) [56] was used to trim artificial sequences (adapters), cut low quality bases (quality scores < 20), and remove short reads (< 50 bp). In addition, Tophat2 (version 2.0.9) [57] was used to remove potential host DNA and RNA contamination by mapping trimmed reads to the bovine genome (UMD 3.1).

### Identification of ARGs using read- and assembly-based approaches

The prevalence and abundance of the rumen resistome of each metagenomic dataset was determined using read-based approach according to the instructions of ARG-OAP 2.0 pipeline [58]. Briefly, post-QC reads (paired-end) from each sample were blasted using BLASTX against the Structured ARG database (SARG) [59], comprised of the Antibiotic Resistance Genes Database (ARDB), the Comprehensive Antibiotic Resistance Database (CARD), and the National Center for Biotechnology Information Non-Redundant Protein Sequence Database (NCBI-NR), to extract ARG-like reads. Reads were subsequently annotated as ARG-like reads at the cut-off of *E* value of 10^−10^, sequence identity of 80% and alignment length more than > 25 amino acids (six-frame translation) using the default settings. By using this cut-off, the identification accuracy was estimated at 99.5% [60]. To evaluate the expression of the resistome, both SARG database [59] and extracted ARG-like sequences identified in metagenomic datasets were used as reference database to extract ARG-like transcripts from the metatranscriptome dataset using an ARG-OAP 2.0 pipeline [58]. Reads were annotated as ARG-like transcripts using the same cutoffs as described above.

In addition, the prevalence and abundance of the rumen resistome was also determined using assembly-based approach. Briefly, metagenomic and metatranscriptomic assemblies were generated with post-QC reads for each sample using MEGAHIT (version 1.2.9) [61] with default parameters. Quality of metagenomic and metatranscriptomic assemblies were evaluated using Quast (http://cab.cc.spbu.ru/quast/) and contigs with lengths less than 1000 bp in all of the assemblies were filtered out. The contigs were then aligned with SARG database using ‘diamond blastx’ [62] at the cut-off of *E* value of 10^−10^ and sequence identity of 80% for both metagenomic and metatranscriptomic sequencing.

### PCR analysis of the prevalence and expression of *tetQ*, *tetW*, and *mefA* genes

All the DNA isolates (50 ng/μl) and cDNA were tested for the presence of antigen resistance genes by PCR. Individual PCRs were performed for *tetW*, *tetQ* and *mefA*. The primers and PCR conditions used were those described in Additional file 3: Table S15. The PCR assays were conducted in a 20-μl mixture containing 2 ul 10 × PCR buffer, 0.5 μl, 10 mM dNTP, 0.25 ul, 5 U/μl of Taq polymerase (Life Technologies, Foster City, CA), 1ul, 20 pmol/μl of each primer and nuclease-free water. Reactions were conducted using an Vetiri 96 well thermal cycler (Life Technologies, Foster City, CA). *TetW*, *tetQ* and *mefA* were amplified by subjecting DNA and cDNA template to the following conditions: an initial denaturation for 5 min at 94℃; then 30 cycles of 94℃ for 30 s, annealing for 30 s at different temperatures (Additional File 1: Table S13), and 72℃ for 30 s; and a final elongation for 7 min at 72℃. Finally, the PCR products were analyzed by gel electrophoresis using 1% (w/v) agarose in 1 × TBE buffer under 150 V. The agarose gel was steamed in Sybr safe and scanned under Azure c200 image system (Azure Biosystems Inc, Dublin, CA).

### Identification of plasmid-associated ARGs

Plasmid-associated ARG were determined using a modified ARG-OAP 2.0 pipeline. Instead of SARG database, post-QC reads (paired-end) from each sample were blasted using BLASTX against the latest A CLAssification of Mobile genetic Elements (ACLAME) database, which contains information of mobile genetic elements including 457 bacteriophage genomes, 1109 plasmids and 760 prophages [63]. Plasmid associated reads and transcripts were then annotated at a cut-off *E* value of ≤ 10^−7^ criteria with amino acid identity ≥ 80% and coverage ≥ 70%.

### Identification of bacterial origin of expressed ARGs

The bacterial origins of the active resistome were predicted by assigning taxonomy to metatranscriptomic-assembled contigs harboring ARGs using kraken2 [64]. First, the complete genomes for the bacterial domain in NCBI RefSeq (ftp://ftp.ncbi.nlm.nih.gov/genomes/refseq/bacteria/) [65] was downloaded with ‘kraken2-build –download-library bacteria –db RefSeq’ script. Then, the 4941 rumen-related metagenome-assembled genomes [66] were added to the RefSeq database with ‘kraken2-build –add-to-library 4941.fa –db RefSeq’ script. The metatranscriptomic-assembled contigs with ARGs were subject to the ‘kraken2 –db RefSeq –threads 32 –classified-out sample_classified –unclassified-out sample_unclassified -confidence 0.9 –fasta-input sample.contigs.fa –output sample.kraken’ script, where ‘RefSeq’ is the customized database, ‘0.9’ is the confidence score (between 0 and 1). To verify the bacterial host of an ARG, using a parallel approach, the full ARG-containing contigs, as well as their counterparts containing the left-over flanking regions following the SARG database BLAST-based removal of ARGs from each contig, were annotated using kraken2. The output files were further analyzed using Bracken [67] with ‘bracken -d RefSeq -i sample.kraken.txt -o sample.bracken.txt -l S’ script to calculate the relative abundance of bacteria at the species level. In addition, the flanking regions were further blasted against Gypsy Database [68] (https://gydb.org/index.php/Blast) to detect potential mobile genetic elements with E-value lower than 0.01.

### Functional profiling of active ruminal microbiome

To analyze the functional profiles of the active ruminal microbiome, metatranscriptomic-assembled contigs were also annotated using Prodigal [69]. The output was then uploaded to KofamKOALA, which assigns K numbers by HMMER/HMMSEARCH against KOfam (a customized HMM database of KEGG Orthologs (KOs)) with an e-value lower than 0.01, to annotate the KEGG functional pathways [70]. The abundance of active KEGG functional pathways was normalized into counts per million.

### Stability of active rumen microbiome

Attenuation value is a measurement of microbial stability, which evaluates the expected rate at which increases in the taxonomic perturbation magnitude are expected to increase functional profile shifts [71]. For example, our previous study showed that the attenuation values of fecal microbiota of neonatal calves increased over age, suggesting an increase in stability is a feature of gut microbiota in early life [72]. The attenuation value was calculated according to the method described by Eng and Borenstein [71]. As this method applies 16S rRNA dataset as input, we extracted 16S rRNA sequences from metatranscriptomic datasets using SILVA database of kraken2 [64]. Then the extracted 16S rRNA sequences were demultiplexed with “demux” plugin and subjected to quality control (QC) using “dada2” plugin [73] of QIIME2 (version 2020.2) [74] to generate a table containing the taxonomic composition of a sample. Finally, varying fold of perturbations of each community’s taxonomic composition was simulated and the average shift in the functional profile of a community as a power function of the taxonomic perturbation magnitude was established as:

$$f= \frac{1}{{e}^{a}}{t}^{b}$$

where *t* indicates the magnitude of simulated taxonomic perturbation, *f* indicates the expected shift in functional capacity, *a* (attenuation) is defined as inversely proportional to the response curve slope, indicating the expected rate at which increases in the taxonomic perturbation magnitude are expected to increase functional profile shifts, *b* (buffering) indicates how large a perturbation must be before a functional profile shift becomes noticeable and approaches the expected shift magnitude defined by attenuation [71]. As the buffering values did not correlate with the diversity or abundance of expressed ARGs, they were not reported.

### Calculations and statistical analyses

The abundance of ARG classes, total ARGs, and individual ARG were calculated as ‘number of reads per million of post-QC metagenomic/metatranscriptomic reads’ or ‘number of assemblies per million of post-QC metagenomic/metatranscriptomic assemblies. Difference in the proportion of post-QC metagenomic/metatranscriptomic reads aligned to bovine genome, the abundance of ARGs and ARG transcripts among breeds were analyzed using the Kruskal–Wallis test in R (version 3.6.1). The *P*-value of multiple comparison of breed effect was adjusted into false discovery rate (FDR) using the Benjamini–Hochberg algorithm using ‘dunnTest’ function in R. The effect size of multiple comparisons was conducted using ‘Cohen’s d’ function in R. Circos plot analysis was performed in R using the RCircos package [75]. Alpha diversity indices, including Chao1 and Simpson’s index, was performed using ‘chao1’ and ‘diversity’ functions in R, respectively. Principle Coordinate Analysis (PCoA) based on Bray–Curtis distance was performed for the ARGs and ARG trasncripts using ‘betadisper’ and the results were visualized using ‘plot’ function in R. Spearman correlation analysis was performed for the abundance of expressed ARGs and RFI, functional profiles, and attenuationn value of active microbiome and the results were visulized using ‘ggscatter’ and ‘ggplot2’ functions in R Studio. The *P*-value of Spearman correlations were corrected for multiple inference using Holm's method using ‘rcorr.adjust’ function in R. Significant difference was declared at *P* ≤ 0.05 and tendencies at 0.05 < *P* ≤ 0.10. The Spearman’s correlation coefficient, known as rho (ρ), ranges from − 1.00 (a perfect negative correlation) to + 1.00 (a perfect positive correlation). A rho value higher than 0.40 or lower than − 0.40 as well as *P* value ≤ 0.05 is considered as significant correlation.

## Supplementary Information

**Additional file 1.** Table S1–S14.

**Additional file 2.** Fig. S1–S8.

**Additional file 3: Table S15.** The primers used for PCR analysis of *tetW*, *tetQ*, and *mefA* genes.

## Data Availability

Rumen metagenome and total-RNA-based metatranscriptome sequences were deposited into NCBI Sequence Read Archive (SRA) with accession number SRS4217479, and SRS4211817-SRS4211863 under bioproject number PRJNA448333.
