# Supplementary figures and images for: Expressions of resistome is linked to the key functions and stability of active rumen microbiome
Source: Anim Microbiome. 2022 Jun 4;4:38. doi: 10.1186/s42523-022-00189-6 (PMC9167530; doi:10.1186/s42523-022-00189-6)

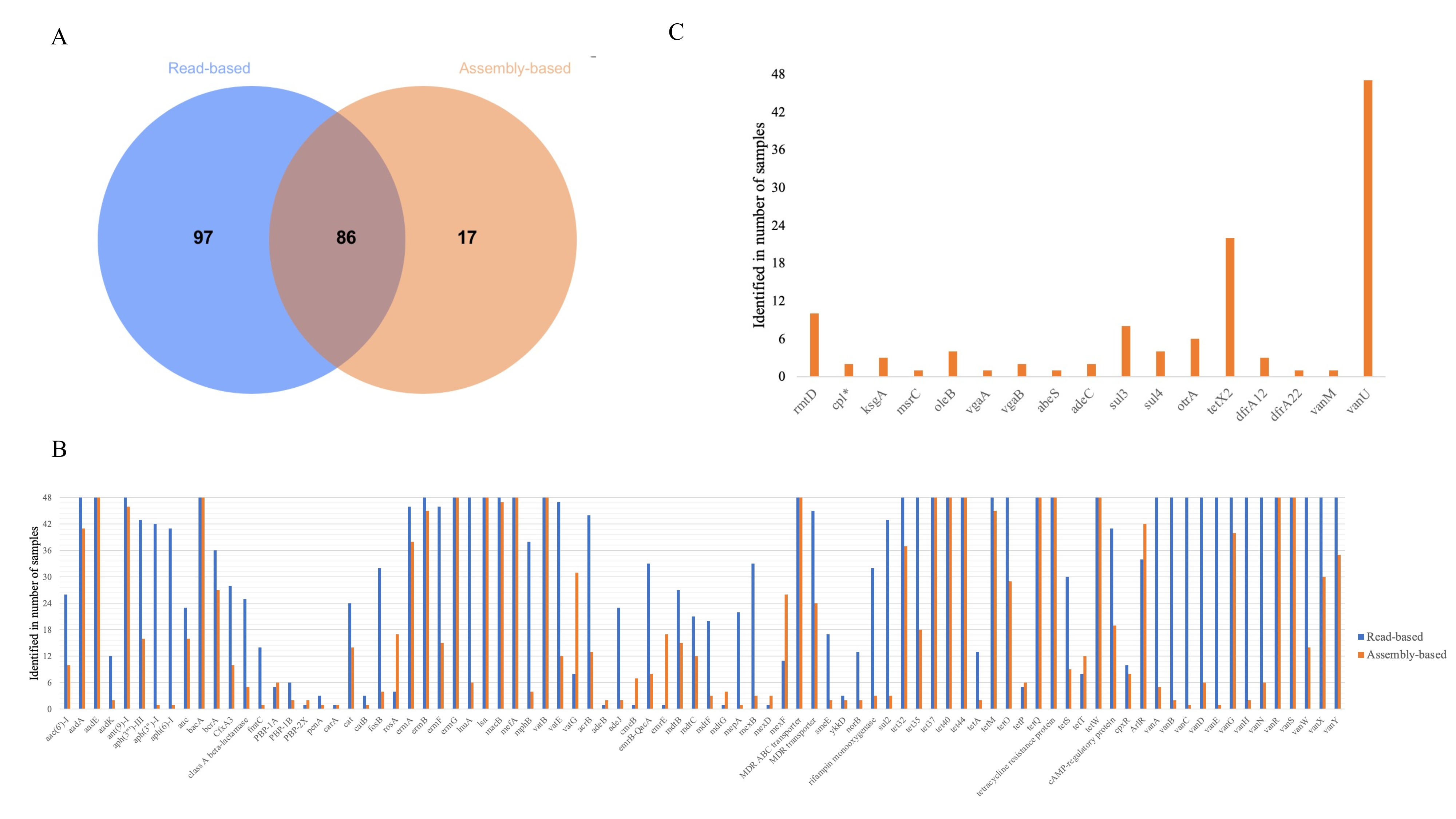

Supplement: Supplementary file 2 — Additional file 2. Fig. S1–S8. [file 42523_2022_189_MOESM2_ESM.zip › 42523_2022_189_MOESM3_ESM/Fig. S1.tif]

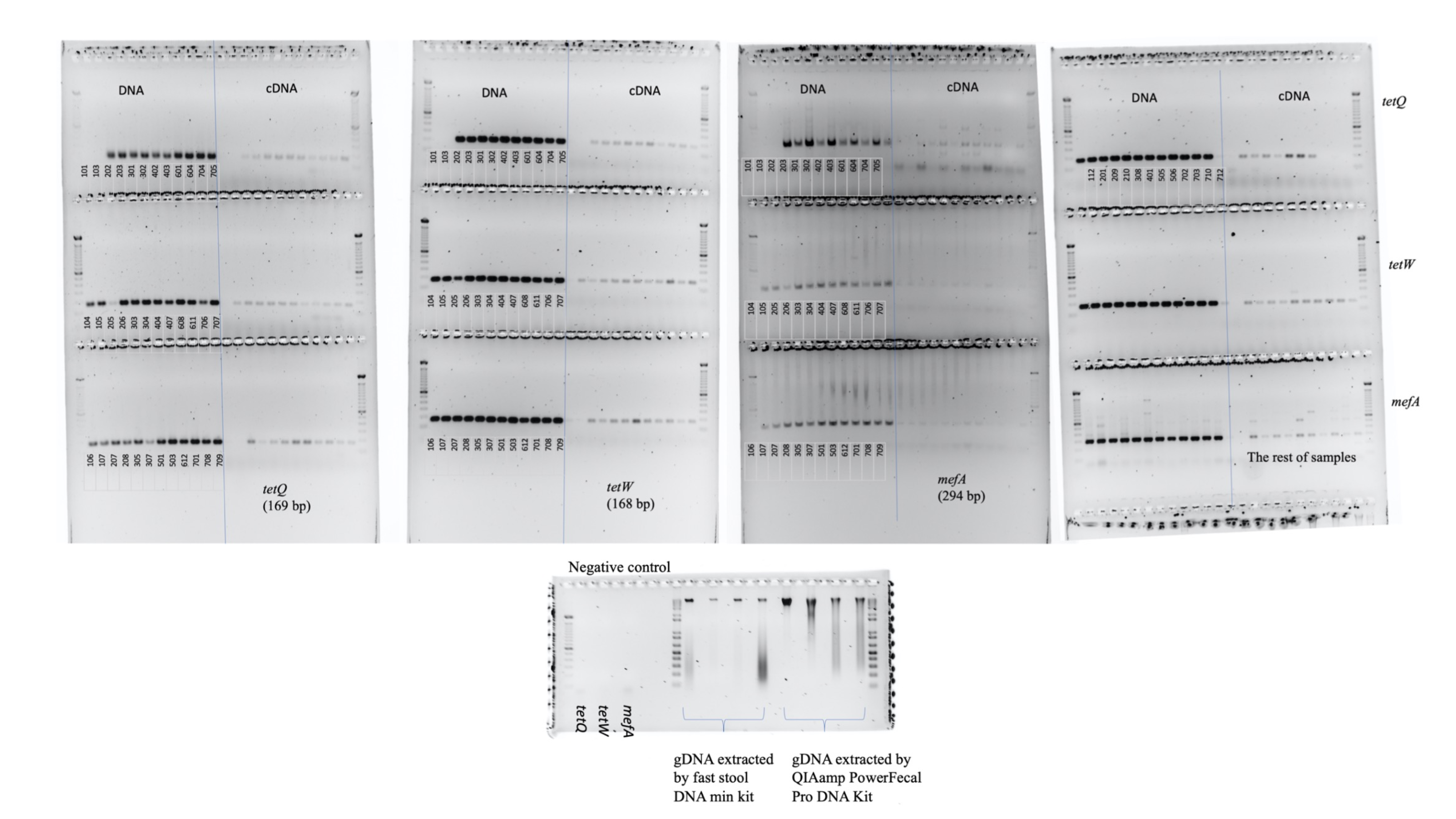

Supplement: Supplementary file 2 — Additional file 2. Fig. S1–S8. [file 42523_2022_189_MOESM2_ESM.zip › 42523_2022_189_MOESM3_ESM/Fig. S2.tif]

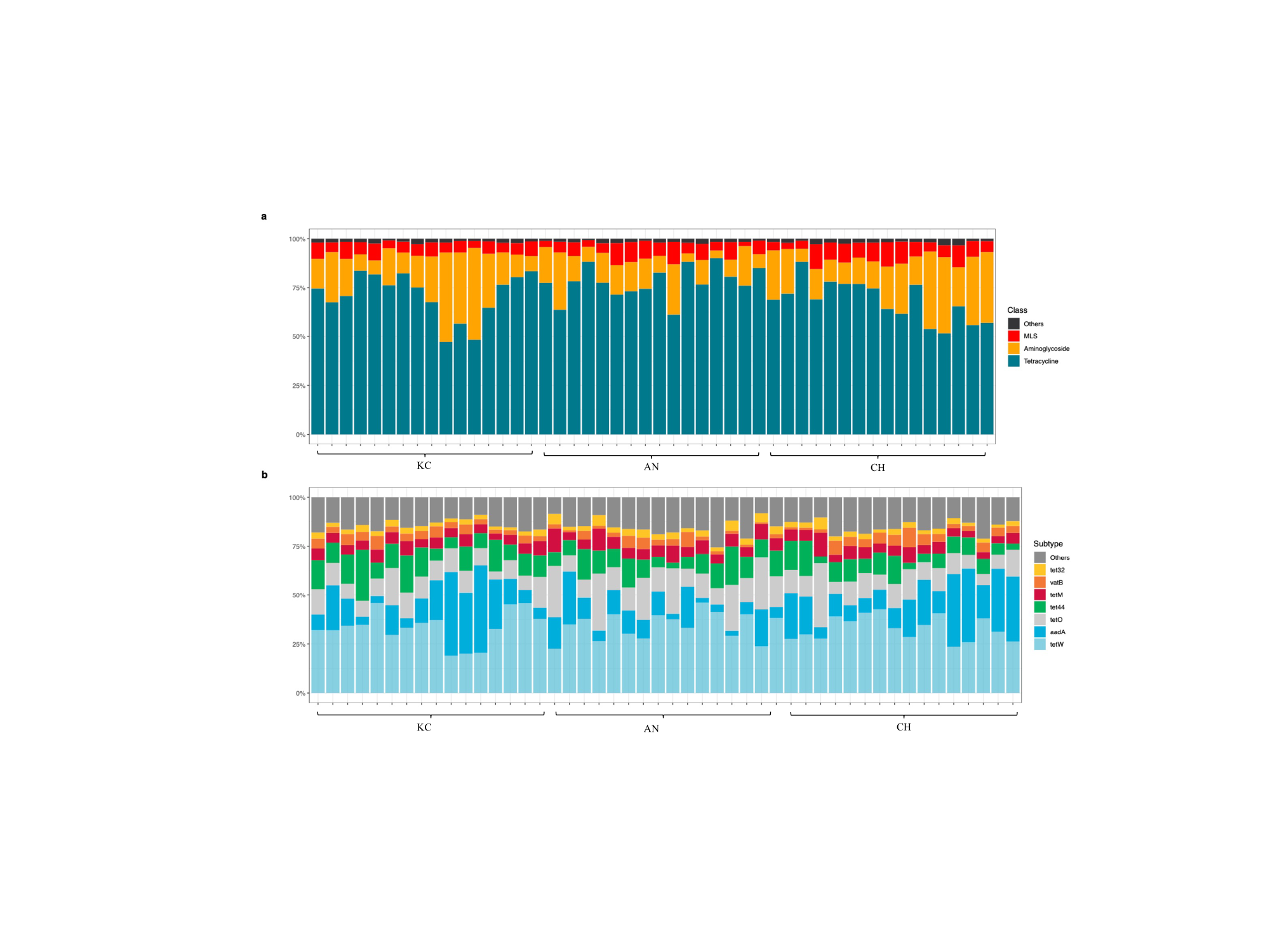

Supplement: Supplementary file 2 — Additional file 2. Fig. S1–S8. [file 42523_2022_189_MOESM2_ESM.zip › 42523_2022_189_MOESM3_ESM/Fig. S3.tif]

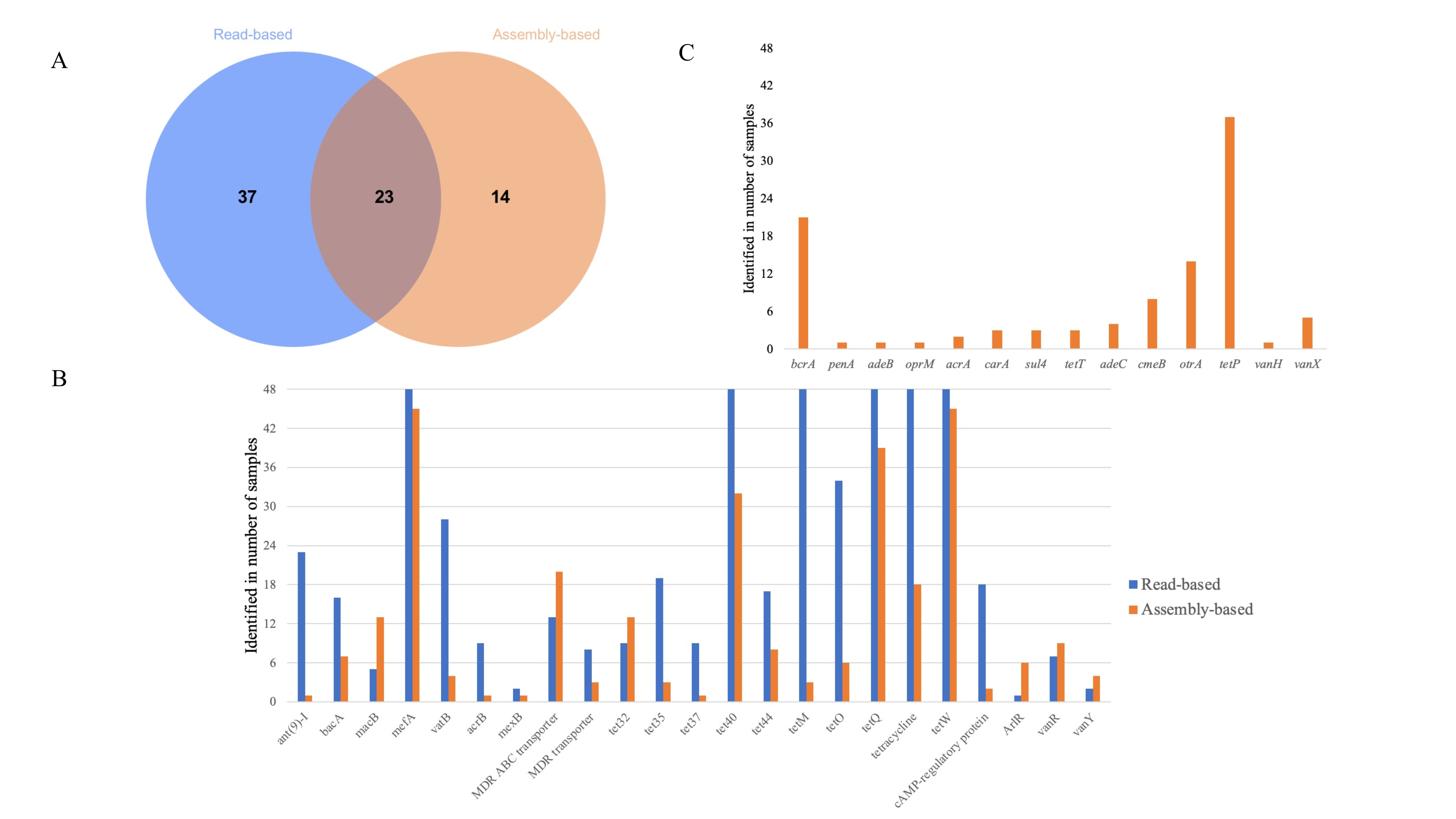

Supplement: Supplementary file 2 — Additional file 2. Fig. S1–S8. [file 42523_2022_189_MOESM2_ESM.zip › 42523_2022_189_MOESM3_ESM/Fig. S4.tif]

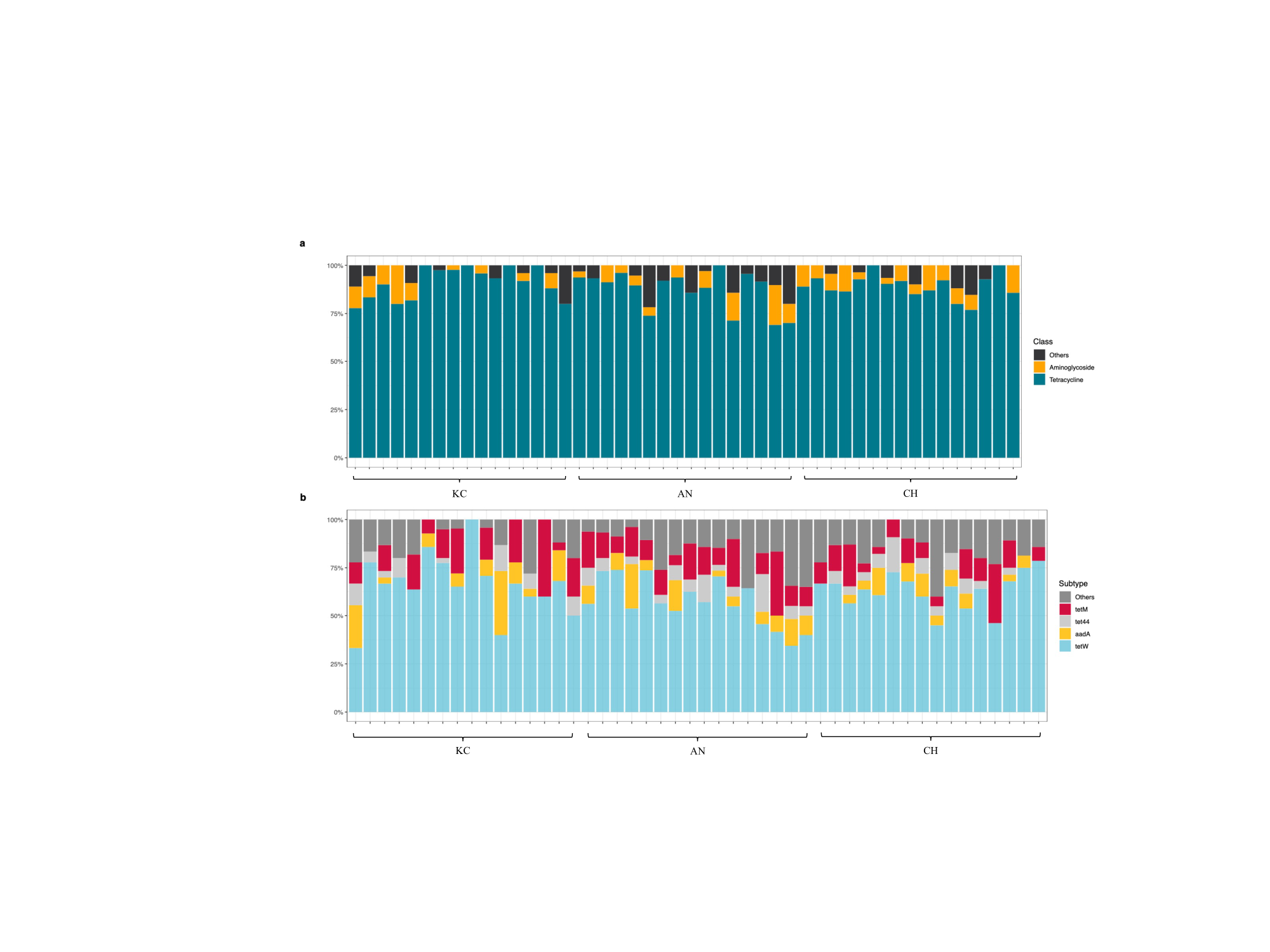

Supplement: Supplementary file 2 — Additional file 2. Fig. S1–S8. [file 42523_2022_189_MOESM2_ESM.zip › 42523_2022_189_MOESM3_ESM/Fig. S5.tif]

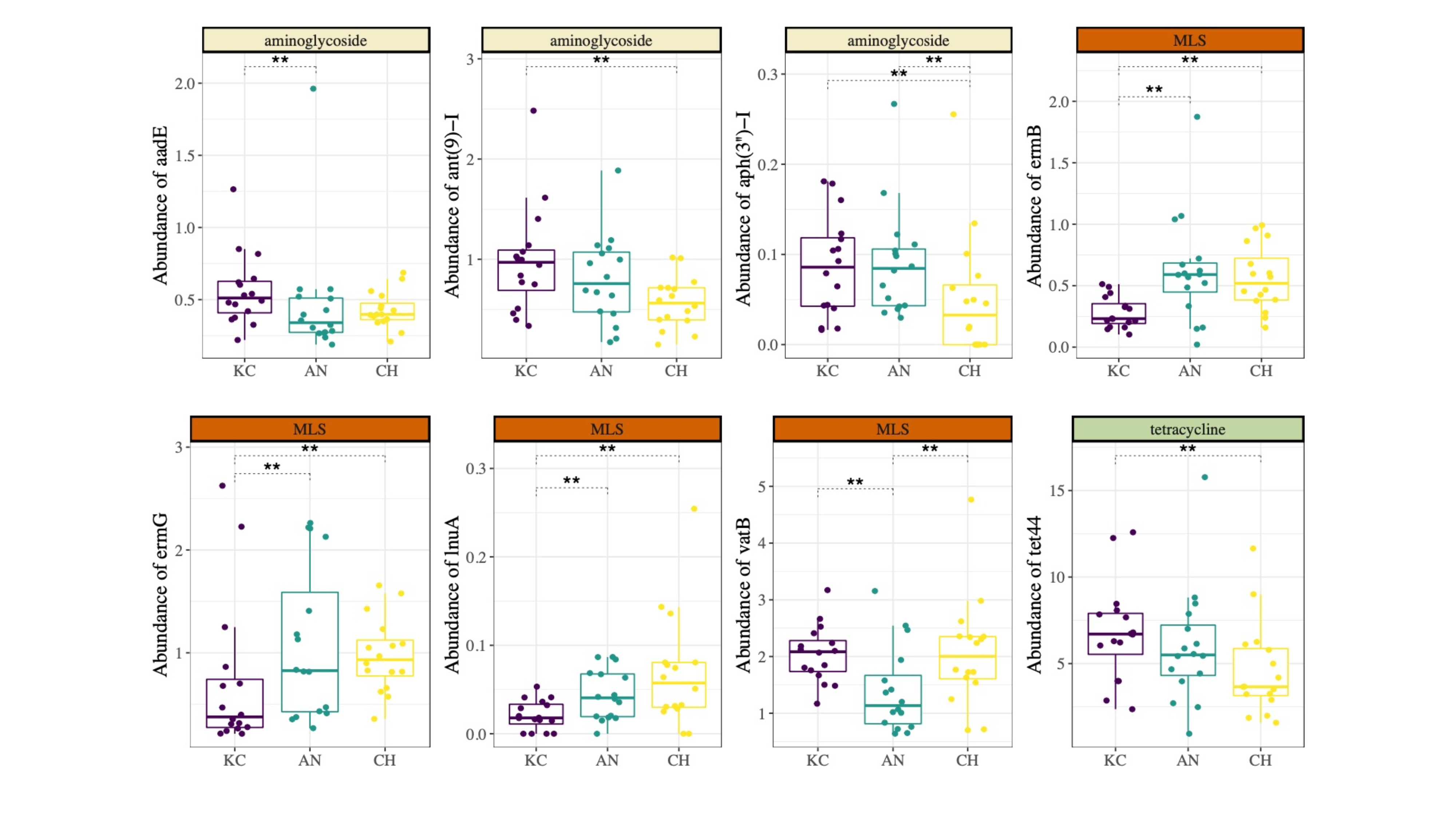

Supplement: Supplementary file 2 — Additional file 2. Fig. S1–S8. [file 42523_2022_189_MOESM2_ESM.zip › 42523_2022_189_MOESM3_ESM/Fig. S6.tif]

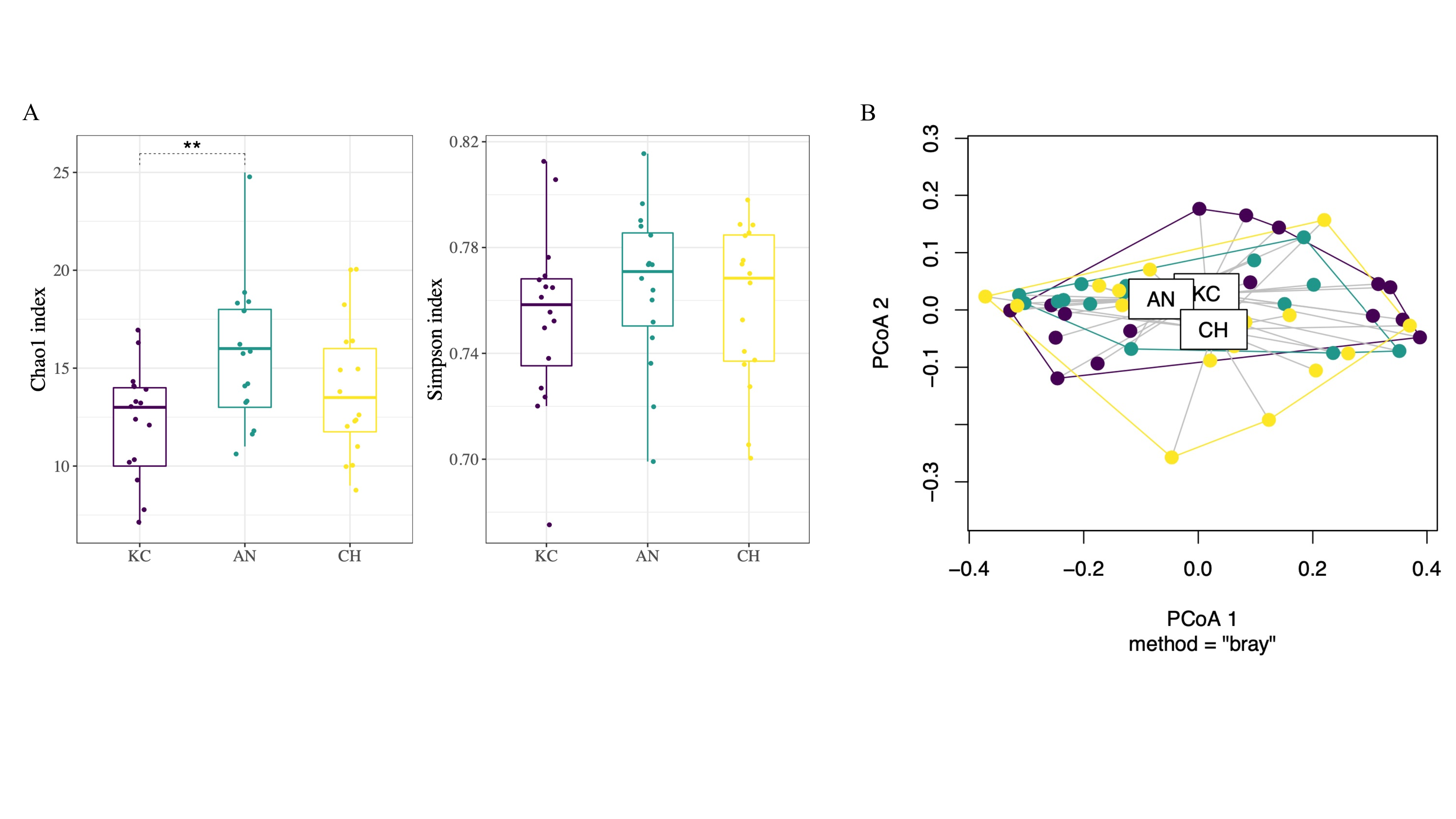

Supplement: Supplementary file 2 — Additional file 2. Fig. S1–S8. [file 42523_2022_189_MOESM2_ESM.zip › 42523_2022_189_MOESM3_ESM/Fig. S7.tif]

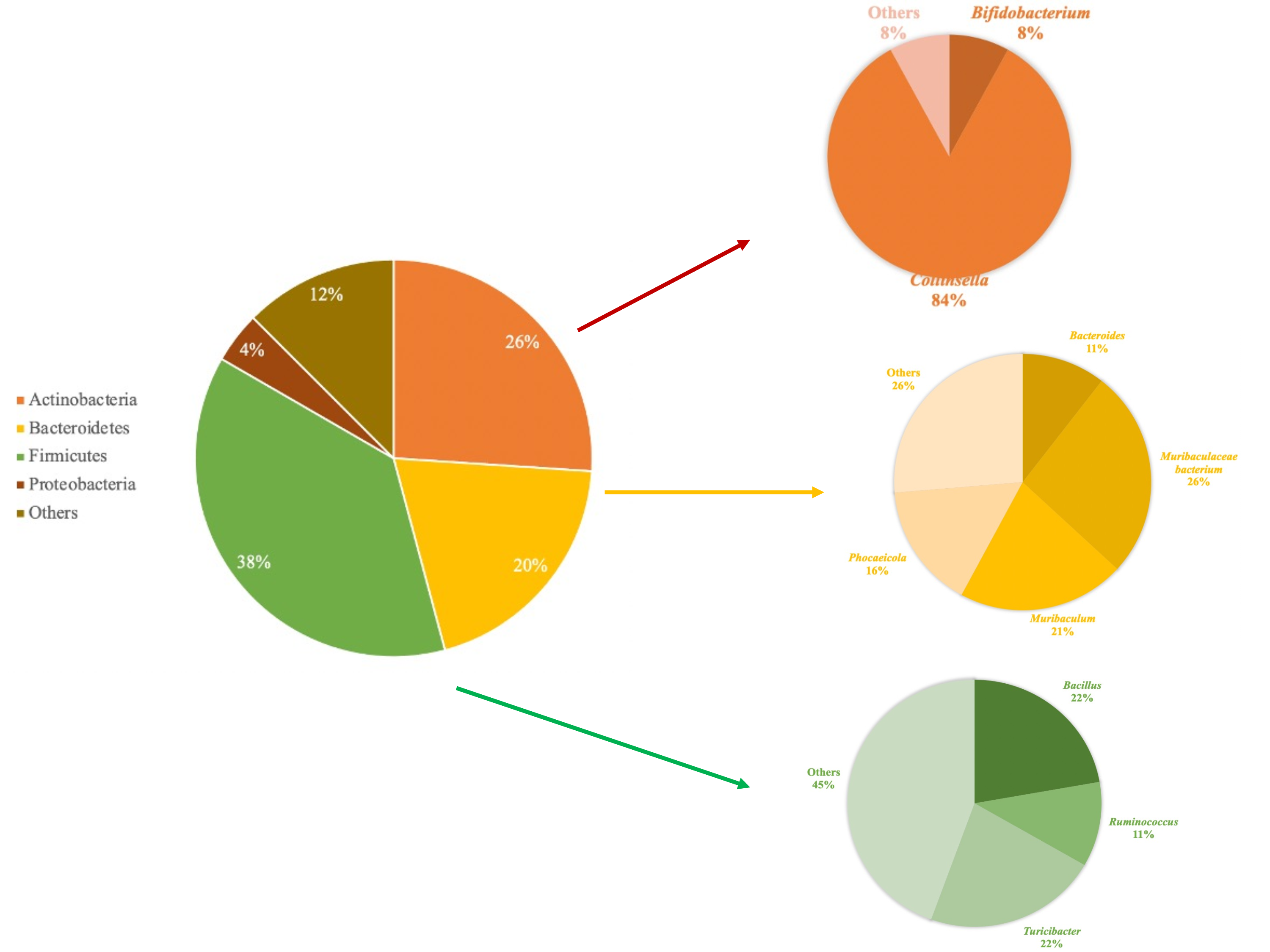

Supplement: Supplementary file 2 — Additional file 2. Fig. S1–S8. [file 42523_2022_189_MOESM2_ESM.zip › 42523_2022_189_MOESM3_ESM/Fig. S8.tif]
